# A wrap-around movement path randomization method to distinguish social and spatial drivers of animal interactions

**DOI:** 10.1098/rstb.2022.0531

**Published:** 2024-09-04

**Authors:** Kaija Gahm, Ryan Nguyen, Marta Acácio, Nili Anglister, Gideon Vaadia, Orr Spiegel, Noa Pinter-Wollman

**Affiliations:** ^1^ Department of Ecology and Evolutionary Biology, University of California, Los Angeles, CA, USA; ^2^ School of Zoology, Tel-Aviv University, Tel Aviv, Israel

**Keywords:** null models, randomization, social network analysis, spatial constraints, animal movement, GPS telemetry

## Abstract

Studying the spatial–social interface requires tools that distinguish between social and spatial drivers of interactions. Testing hypotheses about the factors determining animal interactions often involves comparing observed interactions with reference or ‘null’ models. One approach to accounting for spatial drivers of social interactions in reference models is randomizing animal movement paths to decouple spatial and social phenotypes while maintaining environmental effects on movements. Here, we update a reference model that detects social attraction above the effect of spatial constraints. We explore the use of our ‘wrap-around’ method and compare its performance to the previous approach using agent-based simulations. The wrap-around method provides reference models that are more similar to the original tracking data, while still distinguishing between social and spatial drivers. Furthermore, the wrap-around approach results in fewer false-positives than its predecessor, especially when animals do not return to one place each night but change movement foci, either locally or directionally. Finally, we show that interactions among GPS-tracked griffon vultures (*Gyps fulvus*) emerge from social attraction rather than from spatial constraints on their movements. We conclude by highlighting the biological situations in which the updated method might be most suitable for testing hypotheses about the underlying causes of social interactions.

This article is part of the theme issue ‘The spatial–social interface: a theoretical and empirical integration’.

## 1. Introduction

Animal movement patterns are influenced by both the physical and the social environment. Animals may seek the presence of others because of the benefits they gain from sociality (e.g. removal of parasites, avoidance of predators) [1], or they may avoid each other (e.g. owing to competition). These social interactions are affected by the physical environment because most animals need to be in proximity to one another to interact [2]. The physical environment might facilitate or constrain social interactions—for example, by attracting individuals to a shared resource [3–5] or preventing them from moving across barriers [6,7]. Uncovering whether social interactions are a result of social attraction (social phenotype and social environment) or whether they emerge from spatial constraints on animal movements (spatial phenotype and spatial environment) is important for understanding the function of social interactions and the evolution of sociality, and for predicting how changes in external conditions (e.g. habitat fragmentation) will affect animal societies [8]. Here, we introduce a method that facilitates dissecting the contribution of these two forces in shaping animal interactions measured from tracking data.

The increased availability of high-resolution GPS data on animal movements has opened up new opportunities to examine the relationship between social behaviour and the environment [9–11]. Social interactions are often inferred from the proximity of individuals fitted with GPS tags [12,13]. At the same time, GPS tags provide high-resolution information about animal movements and can be used to examine how these movements are shaped by resources and obstacles in the environment [11]. Such rich movement data allow us to investigate whether social interactions emerge from conspecific social attraction or if they are simply a by-product of the movements of animals within a given spatial environment [8,14].

Testing hypotheses about the drivers of sociality, or the relative contributions of multiple factors, is often based on comparing observed interactions with social interactions generated by reference (or ‘null’) models that carefully account for underlying factors of interest. Because social networks violate many of the assumptions of traditional statistical methods [15], their analysis often relies on the construction of biologically meaningful reference models using various randomization approaches, such as the one we investigate here [16,17]. Many questions in animal social network analysis do not require accounting for spatial information when constructing reference models. For example, asking whether the social role of an individual can be predicted by its attributes, such as age [18,19], sex [20,21] or personality [22,23], can be answered using permutations of node identities that maintain the observed network structure [17]. However, many questions about animal sociality focus on the underlying proximate causes of social interactions (e.g. do animals interact more frequently than by ‘chance’?). To answer such questions, researchers have compared observed interactions to interactions formed by a wide range of reference models. The broadest reference model would compare observed interactions to random networks, in which interactions are drawn from some distribution that may, or may not, be biologically grounded [24]. The less restricted the reference model, the easier it is to reach the conclusion that animals interact non-randomly. Such reference models, like the ideal gas model [25], often neglect the biologically meaningful processes that underlie the formation of social interactions; for example, the influence of environmental features on movements. To determine whether social attraction is a cause of social interactions, or whether interactions result from how the environment shapes animal movements, it is important to construct reference models that account for animal space use patterns while controlling for the effects of their social attraction to each other.

A number of approaches have been proposed for disentangling social and spatial drivers of animal social interactions. Most approaches to this question rely on comparing observed interactions with reference models that randomize the raw movement data before constructing the social network (often called data-stream randomization), rather than shuffling nodes of the social network itself. Randomizing raw movement data allows for the decoupling of social and spatial processes [14,17]. An initial data-stream randomization approach permuted the identities of individuals among movement trajectories [26] or among groups [27], allowing one to ask if particular individuals were more likely than chance to occupy certain social positions, given spatial constraints on animal movements. However, these methods could not determine whether interactions resulted from social attraction or emerged from spatial constraints. Similarly, a recent method [28] can help identify population-level interaction hotspots in the environment. However, this approach does not directly address whether animals arrive at attractors because they are searching for resources, avoiding threats or seeking social encounters.

To address this gap, Spiegel *et al*. [14] introduced a novel path randomization approach that decouples the impact of animal movements from that of social attraction on the formation of interactions. This decoupling is accomplished by randomizing the temporal order of movement paths and inferring interactions from the randomized data. Because this randomization decouples the movements of individuals from one another, while maintaining the impact of the physical environment on the movement shape of each individual, it uncovers how social attraction (or repulsion) affects the formation of interactions in the observed data. We will refer to this method as ‘path shuffling’ to distinguish it from other approaches that randomize movement data-streams. In short, this approach permutes segments of movement paths within an individual’s own trajectory (e.g. by shuffling day-long segments of movement) to decouple the synchronized movements of interacting individuals, while maintaining the spatial component of each individual’s movement patterns. Importantly, this method retains the association between individual identity and explicit use of space, preserving individual variation in movement and space use (i.e. territories, preferences for specific locations, variation in the amount or nature of movement, etc.). Thus, it allows one to identify whether social interactions emerge solely from movement patterns, or whether they arise from social attraction and movement synchrony. Path shuffling also allows for identifying whether the locations of encounters differ from those expected by chance [20]. The path shuffling method has been implemented to understand patterns and drivers of social interactions in cows [29], hyenas [30], colonial seabirds [31], caribou [32] and sleepy lizards [20]. It has been integrated into the widely-used R package ‘spatsoc’ [13], and a modified version also appears in the ‘contact’ R package [33]. The method, conducted on a timescale of days, produces biologically sensible and robust reference models for central place foragers that return to the same location (e.g. a burrow) every night. However, if one is interested in asking questions about animals that frequently move among sleeping locations, the path shuffling approach introduces into the reference models biologically unfeasible ‘teleportations’—situations in which a trajectory of an animal ends in one location on one day, and owing to the path shuffling procedure, continues from a completely different place on the following day. Because reference models should be shaped by the biological question asked and aspire to disrupt the observed data as little as possible, beyond the effect that is being tested, this ‘teleportation’ shortcoming can limit the applicability of path shuffling in some instances, highlighting the need to expand the generality of the approach.

Here, we propose an alternative trajectory data-stream randomization approach that circumvents the teleportation problem. The ‘wrap-around’ method, a modification of the path shuffling method, shifts the entire movement path of each individual forward or backward in time, and wraps the trajectory back to the start (or end) to retain the duration and timing of the observation period (see more details in §2a). This method, like the path shuffling, also breaks the temporal synchrony between individuals while retaining both the within day and day-to-day sequence of movements for each individual. Shifting trajectories instead of shuffling them is not entirely new; a version of the wrap-around method was used by Benhamou *et al*. [34], and a few other studies have also used temporal shifts [35–37]. Still, the wrap-around method has not been widely adopted, despite its greater biological realism than path shuffling. Furthermore, its performance and ability to distinguish between social and spatial drivers of interactions in different situations, compared with the path shuffling method, has not been evaluated. Here, we use agent-based simulations to examine the performance of the wrap-around method, compare it with the path shuffling approach, and apply it to animal data. Importantly, both methods use randomization to decouple spatial and social drivers of interactions by randomizing spatial data along a temporal dimension. However, the degree to which the data are randomized and the amount of temporal autocorrelation within the movement paths that is preserved in each randomization slightly differs.

After introducing the wrap-around method, we evaluate its performance under different movement scenarios using agent-based simulations, and compare it with path shuffling. Specifically, we predict that while the path shuffling and wrap-around methods will perform similarly when animals return to the same place each night, they will differ in their performance when animals change sleeping sites over time. We further predict that as the amount of shifting of the movement trajectories in the wrap-around method increases, the closer the outcome will be to the path shuffling and farther from the observed interactions. We examine whether the path shuffling and wrap-around methods differ in their rate of false positives (likelihood to detect sociality—either attraction or avoidance—when it is not simulated) or false negatives (not detecting sociality when it is simulated); and how these differences are affected by movement patterns (e.g. when migrating or traplining). Furthermore, we investigate whether the number of observations recorded each day affects the ability of the randomization procedures to distinguish between social and spatial drivers of interactions. Finally, in addition to testing the method’s performance with agent-based models (ABMs), we apply both the wrap-around and path shuffling randomizations to determine whether social interactions of free-living wild griffon vultures (*Gyps fulvus*) are driven by social attraction, or if they are a by-product of the vultures' movement patterns.

## 2. Methods

### (a) Implementation of the wrap-around method

**Figure 1.**
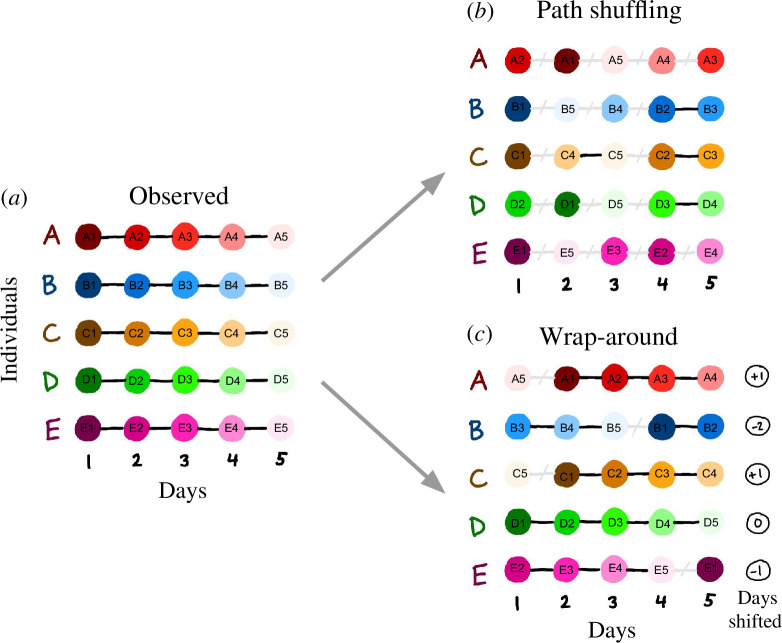
A schematic comparison of the path shuffling and wrap-around randomization methods. Five individuals (A–E) are shown tracked over 5 days, represented conceptually as coloured circles connected by black lines, with hues progressing from dark to light over time (*a*). In the path shuffling method (*b*), the order of days is permuted for each individual, resulting in a large number of ‘teleportation’ events (grey slashed lines). In the wrap-around method (*c*), trajectories are shifted forward or backward by a number of days selected from a uniform distribution between −2 and 2 (the time-shift range), with each individual’s time-shift shown to the right of its trajectory. This method creates a maximum of one ‘teleportation’ event per individual and maintains the order of consecutive days.

In contrast to the path shuffling method, the wrap-around method we propose here shifts animals' movement trajectories forward and backward in time, breaking the temporal synchrony between individuals while retaining the sequence of movements for each individual. Simply shifting each trajectory would expand the total observation period of the randomized data relative to the observed data, and would reduce the density of individuals present on a given day (i.e. simultaneously tracked individuals will seem to have been tracked during different periods). Therefore, when a shifted trajectory reaches the end of the individual’s observation period, we take the remaining days in the trajectory and attach them back to the start of the trajectory, effectively ‘wrapping’ the trajectory around like a conveyor belt. We note that this approach produces a more biologically plausible randomization compared with ‘path shuffling’ because, with one exception (the juncture between the first and last days of the original trajectory), path continuity between days is maintained. As a result, animals do not ‘teleport’ to a new location each night, whereas path shuffling may generate teleportations on a daily basis (figure 1).

### (b) Agent-based model description

To validate our method, we developed a series of ABMs, representing different types of animal movement. These ABMs allowed us to apply the randomization method to a population with known social attraction rules, and to quantify the methods’ ability to detect the underlying social structure (which is unknown in real datasets), or the rates of false detections of sociality. In each model, agents moved in discrete time steps according to a biased-correlated random walk (BCRW), with the direction of each movement step determined by a Von Mises distribution [38]. To represent animals’ tendency to confine their movements to a home range area, we incorporated randomly chosen bias points to simulate ‘home range centres’. The Von Mises distribution has two parameters that determine step direction and concentration. The concentration parameter was chosen to give a semi-linear path similar to that of real animal movements, while agents’ step directions were biased towards their home range centre for the current day. Step lengths were drawn from a gamma distribution with a mean of 7 and a standard deviation of 5—set to produce trajectories that seemed similar to animal movements.

**Figure 2.**
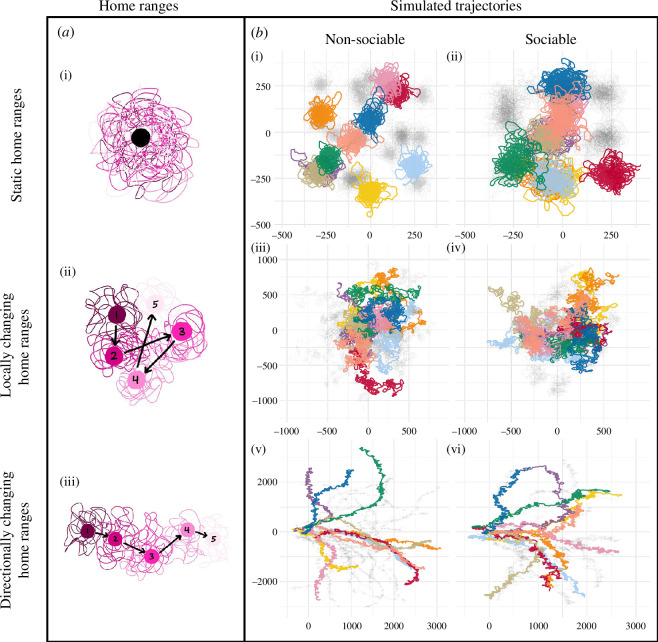
Examples of movement trajectories of home ranges and agents in the ABM. The home range centres to which agents are attracted are either static (*a*(i); *b*(i), *b*(ii), changing locally (*a*(ii); *b*(iii)*, b*(iv)), or changing in a directional manner (*a*(iii); *b*(v)*, b*(vi)). In (*a*(i)), the home range centre is shown as a large black circle and in (*a*(ii), (iii)) the numbers on the large circles of home range centres represent five days. Lines are the walking trajectory of an agent attracted to these home range centres, with line colour corresponding to the colour of each day’s home range centre. The movements of each agent in the ABM are based on correlated random walks in which individuals are either non-sociable (*b*(i), (iii), (v)) or sociable and attracted to their nearest neighbour (social weight = 0.7, *b*(ii), (iv), (vi)) when home range centres are static (*b*(i),(ii)), changing locally (*b*(iii), (iv), or changing in a directional manner (*b*(v), (vi)). In (*b*), the walking trajectories of 10 randomly selected agents throughout the 50 days of a single simulation are shown in colour and the 20 trajectories of the remaining individuals are in light grey. Note the different spatial scales across (*b*(i)-(vi)).

To explore whether the path shuffling and wrap-around methods differed in their performance more when animals displaced farther from their starting points each day, we varied the location of each individual’s home range bias point to create three scenarios (figure 2*a*). In the ‘static home ranges’ scenario, each individual’s bias point was held constant throughout the duration of the simulation. In the 'locally changing home ranges’ and ‘directionally changing home ranges’ scenarios, the bias point of each individual followed its own BCRW, with the direction of bias point movement chosen from a uniform (for ‘locally changing’) or a heavily concentrated (for ‘directionally changing’) Von Mises distribution. These different distributions led to either highly tortuous or relatively linear bias point movements, resulting in changes to the home ranges and to the individuals' tracks over the course of the simulation (figure 2*b*). Bias point movement step sizes were drawn from a gamma distribution with mean equal to the agent step size multiplied by either 0.01 (static home ranges scenarios) or 10 (changing home ranges scenarios) and a standard deviation of 0.75 times the mean home range step size. These values were selected to produce trajectories that approximated the movement patterns that we were interested in investigating.

For each of the above three scenarios, we compared ‘non-sociable’ and ‘sociable’ agents (figure 2*b*) to examine whether the shuffling and wrap-around differed in their false positives (detecting sociality when it was not simulated) or false negatives (not detecting sociality when it was simulated). Non-sociable agents were indifferent to others and did not adjust their movements according to other agents in the simulation. Sociable agents could perceive other agents within a certain ‘social perception distance’ (set to 1000 units) and bias their step direction towards the nearest perceived individual. The starting positions of the agent home range centres were chosen randomly from a square with a side length of two-thirds of the social perception distance—thus, all individuals could perceive each other initially. In our simulations of sociable agents, the agents biased each of their steps towards the weighted average between their home range centre bias point and the position of the nearest perceived conspecific. The relative impact of the nearest neighbour on the movement direction of an agent (social weight) is a tunable parameter, which we set at 0.75 (heavier bias towards a conspecific versus towards the home range centre). To evaluate the performance of the randomization methods at different levels of social attraction, we re-ran the ‘sociable agents’ simulations while varying the social weight between 0.1 and 1 in steps of 0.1. All simulations, analysis and data visualization was conducted in R v. 4.3.1 [39], using the tidyverse packages for data wrangling [40]. The full R code is available on Github (https://github.com/Collaborative-Vulture-Work/Vulture-Conveyor-Belt).

We ran each simulation scenario with 30 agents over 50 days with 50 movement steps per day. We considered one run of the simulations to be the ‘observed’ movements’ and used these movements to determine the observed interactions of the agents. Two individuals were considered to be interacting at a given observation time point if they were closer to each other than twice the mean agent step length (14 units). (For a comparison of this ‘co-location’ definition of an interaction with a more restrictive ‘co-movement’ definition, in which individuals were only considered to be interacting if they were close to each other for two consecutive time steps, see the electronic supplementary material, figures S4 and S5.) We then aggregated these interactions to construct proximity-based weighted social interaction networks. We calculated the degree (number of unique individuals an agent interacted with) and strength (sum of the weights of all social ties) of each agent.

### (c) Randomizations and analysis

To compare the performance of the shuffling and wrap-around randomization methods, we conducted 100 iterations of each randomization method. For the path shuffling method, we randomized dates using the ‘trajectory’ method of the ‘randomizations’ function in spatsoc [13]. For the wrap-around method, individual trajectories were shifted forwards or backwards by a positive integer *s*, for a total ‘time-shift range’ of 2 s (so if *s* is 3 days, then each trajectory may be shifted by a number of days drawn from a uniform distribution between −3 and +3). We note that the power of the method is constrained by *s* (a very small *s* implies that more individuals will maintain their original synchrony in the shifted dataset). Thus, to determine the impact that the time-shift range might have on the performance of the wrap-around randomization method, we ran wrap-around randomizations at each value of *s* between 0 and 25, resulting in time-shift ranges between 0 and 50 days (0–100% of the entire simulation duration). For each randomization iteration, we identified interactions and quantified the degree and strength of each agent, as explained above.

Randomization methods were considered to have succeeded in detecting sociality (i.e. a true positive) if the observed population mean value of degree or strength was significantly more extreme than the population mean values of the randomizations. To quantify the size of the difference, we calculated *Z*-scores for the observed values (electronic supplementary material, tables S1–S3), similar to the method described in [41]. To determine each method’s likelihood of falsely detecting sociality, we examined only the non-sociable simulations. We were interested in cases where the observed agents interacted significantly more or less (or with more or fewer others) than would be expected by chance according to the randomizations. Specifically, we defined false positives of sociality as cases in which we would conclude, based on the difference between the observed data and the randomizations, that social attraction or avoidance existed in the population, when in fact none was simulated. To determine the false-positive rate, we calculated the proportion of the randomization iterations for which the population mean (degree or strength) was either greater than or smaller than the top or bottom 2.5% of the simulation runs to obtain a two-tailed *p*‐value (since we were interested in cases where the observed sociality was either significantly greater than *or* significantly less than expected by the randomizations). To determine each method’s likelihood of failing to detect sociality where it existed (i.e. false negatives), we examined simulations with sociable agents at varying levels of social weight. We were interested in cases where the observed agents did not interact significantly more or less than would be expected by chance according to the randomizations. To determine the false-negative rate, we first obtained a two-tailed *p*-value for detection of sociality, as described above (true-positive rate), and subtracted that from 1 (electronic supplementary material, figure S3).

Because animal tracks are often limited in their observation frequency or have incomplete observations, we examine the effect of observation frequency (i.e. sampling effort) on the false-positive rate. We downsampled the non-sociable 'observed' simulation data for each home range scenario from 50 points per simulation ‘day’ to 25, 10 or 5 points per day. Owing to computational constraints, we ran 50 iterations of each randomization (path shuffling, and wrap-around with time-shift ranges between 4% and 100%). We constructed proximity-based social networks for each randomization, and calculated the false-positive rate for each sampling frequency, type of randomization and time-shift range separately.

### (d) Vulture system

To demonstrate the applicability of our proposed method, we applied it to empirical data from a population of free-ranging Eurasian griffon vultures (*G. fulvus,* hereafter ‘griffon vultures’) in the Negev Desert. The study system has been extensively described in Acácio *et al*., Anglister *et al*., and Sharma *et al*. [42–44]. Griffon vultures are large soaring fliers and obligate scavengers [45]. They travel long distances to feed on ephemeral and widely distributed carcasses, relying on thermal and orographic uplift to save energy in flight. They fly the most during the summer and autumn, when the weather is warm and favourable for thermal soaring. Griffons interact frequently with conspecifics in several social situations [44]. In flight, they rely on visual social cues to locate carcasses [46, 47] and often fly in proximity to conspecifics for extended periods of time [48]. As such, their interactions have the potential to be driven by both conspecific social attraction (or avoidance) and by the distribution of resources on the physical landscape (such as food or uplift). While individual griffons vary in their use of space, they do not consistently return to the same roost every night. Individuals may prefer certain central roost locations, but they alternate between roost locations fairly frequently, even when their daily movements are within the same general area. This makes the vulture dataset particularly suitable for testing how the randomization methods will perform on animals that are not strict central-place foragers.

For this analysis, we focused on data collected from 75 GPS-tagged vultures during the summer of 2022 (15 May–15 September) which makes up approximately 70% of the total population in the area at that time. The GPS tags transmit the location of the vultures approximately every 10 min, providing us with detailed information about their movements (electronic supplementary material, figure S1). We constructed proximity-based social networks of in-flight interactions, to focus on interactions that emerge from continuous movement. Consistent with previous research on this system [44], we defined an in-flight interaction as two individuals flying (i.e. moving faster than 5 m s^−1^) within 1 km of each other during two consecutive 10 min time intervals (to avoid counting simple path-crossings as interactions). We constructed social networks based on these interactions over the duration of the summer. The weight of the edge between each pair of individuals was represented as a simple ratio index—how frequently two individuals were observed interacting, out of the total number of time periods when they were both tracked and possibly could have been observed interacting [49–51]. We calculated each individual vulture’s degree and strength.

We randomized the individual vultures’ movement trajectories according to the path shuffling and wrap-around randomization methods, in units of days. The total length of the summer was 124 days. We tested two time-shift ranges for the wrap-around method, allowing individual trajectories to shift within either a 24 day window (up to ±12 days shift, approximately 20% of the total tracking period) or a 2 day window (up to ±1 day shift, approximately 2% of the total tracking period). Individual vultures were tracked for 121 days on average (range: 60–124 days; s.d.: 9.22 days), had an average daily path length of 118 km (range: 0.07–404 km; s.d.: 72.1 km), and had an average daily displacement (distance between the first and last point of the day) of 17.1 km (range: 0–237 km; s.d.: 26 km). We conducted 100 iterations of each randomization, and then constructed proximity-based social networks from each iteration of the randomized data. We compared the degree and strength of each individual in the observed co-flight social network with its value in the networks constructed from randomized data for each method.

## 3. Results

### (a) Comparing path shuffling to the wrap-around randomization using agent-based models

**Figure 3.**
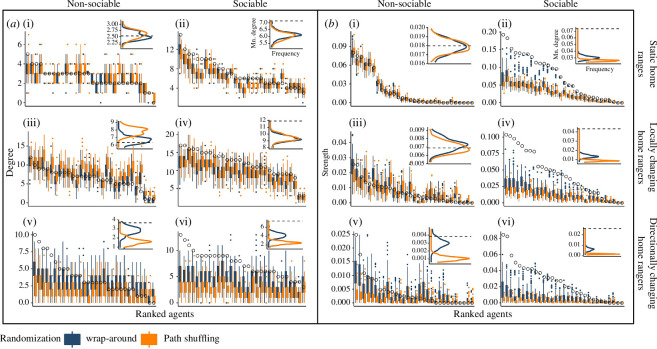
Comparing the path shuffling and wrap-around randomization methods using ABMs. In each plot, agents are ordered by their observed value of degree (*a*) or strength (*b*) (open circles), which are shown on the *y*-axes. Agents are either non-sociable ((i), (iii), (v)) or sociable ((ii), (iv), (vi)) and their home range centres are static ((i), (ii)), change locally ((iii), (iv)) or change in a directional manner ((v), (vi)). The degree or strength values of each agent from 100 iterations of the shuffled (orange) or the wrap-around (blue) randomizations (with a maximum time-shift of 10 days, or 20% of the simulation duration) are shown as boxplots which range to the 25 percentile, with whisker lengths as 1.5 times the interquartile range and outliers as small points. The inset in each panel shows the distribution of degree (*a*) or strength (*b*) values for all agents in all 100 simulation iterations of the shuffled (orange) or the wrap-around (blue) randomizations; the dashed black lines are the average degree or strength values of the ‘observed’ agents.

Both randomization methods detected sociality in all cases where it was simulated (*figure 3a,b*(ii),(iv),(vi) ; electronic supplementary material, figure S3). However, the wrap-around randomization often resulted in values that were closer to observed, compared to the path shuffling method (figure 3*a*(vi)*; b*(ii),(iv),(vi)). Furthermore, the more the location of the home range centre of the animals changed, the larger the difference was between the two methods, as predicted. When home ranges were stationary, there was very little difference between the expected values generated from the wrap-around method and the path shuffling method, especially for degree (figure 3*a*(i),(ii)) and only a slight difference for strength (figure 3*b*(ii)). When home ranges changed locally, the values from the wrap-around method were more similar to the observed values than were the path shuffling values (figure 3a(iii); 3*b*(iv)), or the two methods were nearly identical (figure 3*a*(iv); 3b(iii)). Finally, when home ranges changed in a directional manner, the difference between the two randomizations was the greatest, with the wrap-around values being substantially closer to the observed values relative to path shuffling randomization (figure 3*a,b*(v, vi)).

When social weight was high (agents more strongly biased towards nearby conspecifics than to their own home range centres), none of the randomization methods failed to detect sociality; population mean degree and strength values differed significantly from the population means of the randomizations (electronic supplementary material, figure S3). The wrap-around method, when trajectories were allowed to shift over the entire simulation duration, closely resembled the path shuffling method in its likelihood of failing to detect sociality, and was even more sensitive than the path shuffling method at low levels of sociality in the locally changing home ranges scenario (electronic supplementary material, figure S3*c*). When used with a very small time-shift window, the wrap-around method did have a slightly elevated likelihood of returning a false-negative result at lower levels of sociality, especially for degree in the static home ranges scenario (electronic supplementary material, figure S3*a*).

When simulating non-sociable agents, both methods did not detect sociality when home ranges were stationary (figure 3*a,b*(i)). When home ranges centres changed locally, both methods slightly over-predicted both degree and strength (figure 3*a,b*(iii)); the small difference between the two methods is discussed later in the section about false positives. Finally, both randomization methods detected ‘false-positive’ sociality when home ranges changed in a directional manner (figure 3*a,b*(v)). As predicted, this false-positive detection of sociality was more apparent when using the path shuffling method than when using the wrap-around method, which came closer to correctly identifying that the observed values were generated by a non-sociable process. We explore this further in the section below about false positives.

### (b) Effect of time shifts on wrap-around randomization performance

**Figure 4.**
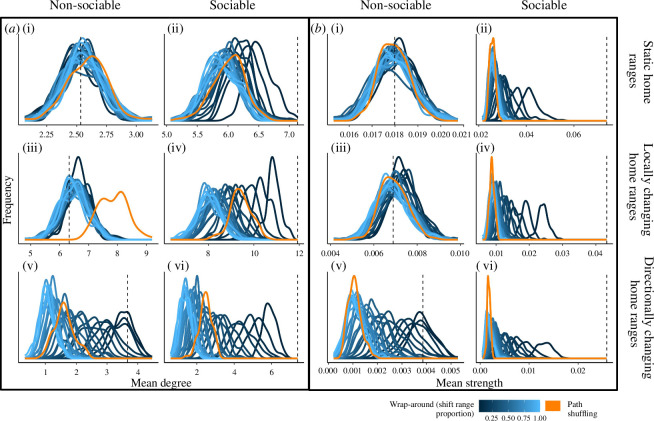
Comparing different time-shift ranges of the wrap-around randomization using ABMs. Distribution of population mean degree (*a*) and strength (*b*) values from 100 randomization iterations when agent’s movement trajectories are shuffled each day (orange lines) or when trajectories are shifted using the wrap-around method (blue lines). Each blue line represents a different allowed time-shift range, as a proportion of the total simulation duration. The proportion of shifting ranges from 4% (darkest blue; as much as ±1 day out of 50 days) to 100% (lightest blue; as much as ±25 days out of 50 days). Dashed lines are the 'observed' mean degree or strength of the population of simulated agents, which are either non-sociable ((i), (iii), (v)) or sociable ((ii), (iv), (vi)). The agents' home range centres are static ((i), (ii)), change locally ((iii), (iv)), or change in a directional manner ((v), (vi)). For a visualization of just the means of the distributions shown here, see the electronic supplementary material, figure S2.

The size of the time-shift range affected how similar the wrap-around randomization results were to the observed values. The shorter the time-shift range, the more similar the randomized data was to the observed data (darker blue lines in figure 4). Note that the wrap-around method results shown in the figure 3 boxplots are for a 20% (10 day) time-shift range, selected arbitrarily from the time-shift range values that we tested. Despite the similarity of short time-shift ranges to the observed data, even the shortest time-shifts still detected sociality—in all scenarios and both for degree and strength (in both figure 4*a,b*(ii),(iv),(vi)). Moreover, when home range centres were changing in a directional manner, only the smallest time-shift ranges avoided false positives for sociality (i.e. only the darkest blue curves overlapped with the dashed line representing observed degree or strength in both figure 4*a,b*(v)).

The time-shift range of the wrap-around method impacted its performance relative to the path shuffling method. For strength, in all scenarios, larger time-shift ranges gave strength values that were more similar to the path shuffling method than those yielded by smaller time-shift ranges (figure 4*b*). When paths were allowed to be shifted over their entire movement range (100% shift proportion, lightest blue lines in figure 4), the strength values returned by the wrap-around method were very similar to those from the path shuffling method (orange lines in figure 4). However, for degree (figure 4*a*), the path shuffling method tended to be most similar to intermediate time-shift range values.

### (c) Sampling frequency and false positives

**Figure 5.**
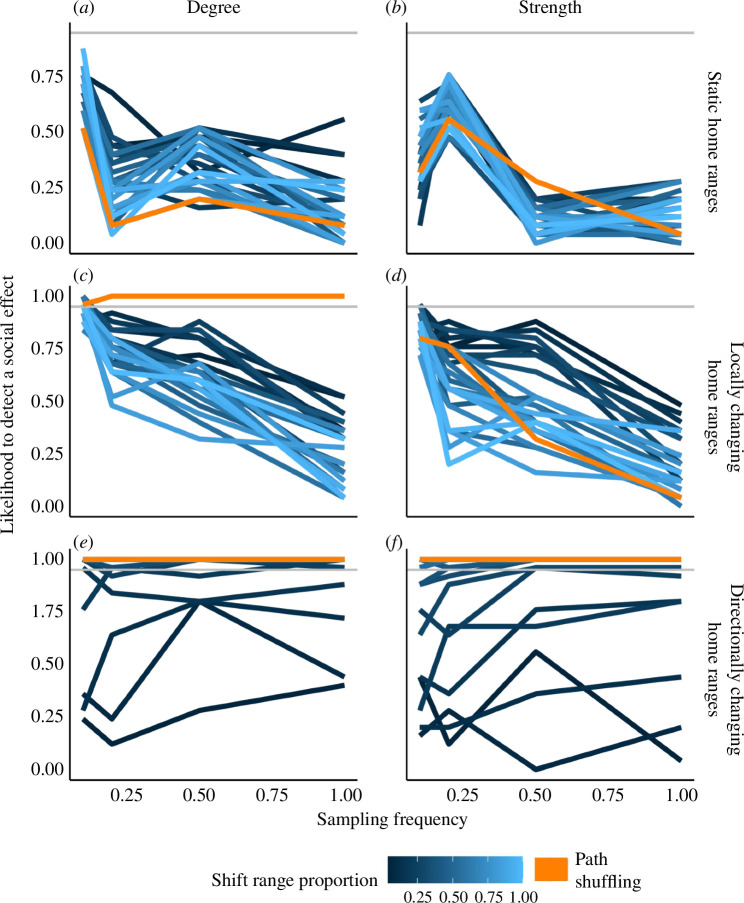
Effect of sampling frequency on the false-positive rate. The likelihood of detecting a social effect (social attraction or avoidance) in the non-sociable simulations, i.e. when there was no underlying social attraction simulated, for the two randomization methods, different time-shift ranges and four sampling frequencies (10%, 20%, 50% and 100% of observed points). Owing to computational constraints, observed values are compared with 50 randomization iterations in this figure. Path shuffling randomization is in orange and blue lines are for the wrap-around method, with shades of blue indicating time-shift ranges, with the proportion of shifting ranging from 4% (darkest blue) to 100% (lightest blue), analogous to figure 4. The grey horizontal lines represent a 95% likelihood (*p* = 0.05). Coloured lines that go above this grey line indicate that a randomization detected sociality even though a social process was not simulated—i.e. a false positive. Panels on the left show false positives for degree, and panels on the right show false positives for strength. Home range centres are static (*a, b*), change locally (*c, d*), or change in a directional manner (*e, f*).

The likelihood of a method to falsely detect sociality was affected by sampling effort (or proportion of missing data), differed between randomization methods, and was influenced by the size of the time-shift range in the wrap-around method. In general, as sampling frequency decreased, the false-positive rate tended to increase, as seen by the negative trends of the lines in figure 5*a,c,d* Interestingly, sampling frequency had less of an effect when home ranges changed in a directional manner (figure 5*e,f*). The path shuffling method showed a weaker relationship between sampling frequency and likelihood of detecting sociality in non-sociable simulations than did the wrap-around method (figure 5*c,e,f*). However, the path shuffling method also detected significantly higher or lower values of degree than expected by chance in both of the non-sociable changing home ranges scenarios (figure 5*c,e*), and more extreme values of strength than expected by chance in the non-sociable, directionally changing home ranges scenario (figure 5*f*). Neither randomization method detected false-positive social attraction in the stable home ranges scenario (figure 5*a,b*). For the wrap-around method, the size of the time-shift range affected the method’s likelihood of detecting sociality. When home ranges changed locally, the wrap-around method returned false positives only at small time-shift ranges (figure 5*c,d*). But when home ranges changed directionally, the method returned false positives, especially at large time-shift ranges (figure 5*e,f*), more similar to the path shuffling method.

### (d) Applying shuffling methods to data from free-ranging vultures

**Figure 6.**
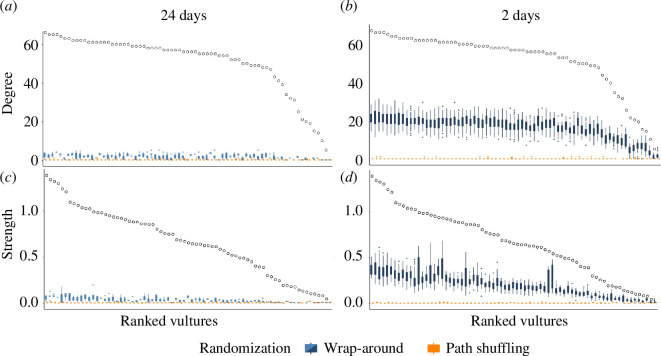
Vulture interactions emerge from social attraction rather than from spatial constraints. Number of unique individuals a vulture interacts with—degree (*a, b*) and number of interactions—strength (*c, d*) of free living vultures during summer 2022 (black open circles). Individual vultures are ordered along the *x*-axis by their observed degree (*a, b*) or strength (*c, d*). The observed values are compared with the degree (*a, b*) and strength (*c, d*) expected by chance when shuffling the trajectory of each day (orange) and when shifting trajectories up to 12 days in either direction (a total time-shift range of 24 days) in (*a, c*) and by 1 day in each direction (total time-shift range of 2 days) in (*b, d*) using the wrap-around method (blue).

When comparing the interactions of vultures with both path shuffling and wrap-around reference models, we found that all individuals had more interactions (both degree and strength) than expected by chance (figure 6). While both randomization approaches resulted in substantially lower social interactions than observed, the wrap-around method produced degree and strength values that were closer to the observed values than the path shuffling method (applied for the entire period). A time-shift range of 24 days (figure 6*a,c*) yielded a much greater difference between the observed and expected values of both degree and strength than did a 2 day time-shift range (figure 6*b,d*), but even this small time-shift range was sufficient to distinguish observed patterns of social interaction from the randomization.

## 4. Discussion

In their paper introducing the path shuffling method, Spiegel *et al*. [14] suggested that ‘A parallel randomization approach that offsets the entire track of a given individual by a varying period… should achieve similar performance*’*. They noted that such an approach should be developed and analysed—as we do here. The existing path shuffling method and the new wrap-around randomization method performed similarly when the agents in our simulation had static home ranges. However, as the agents ranged over larger areas (more mobile home range centres) and changed their foci of activity, the two randomization methods gave more and more different predictions for rates of ’chance’ social encounters (figure 3). When the movement trajectories in the wrap-around method were allowed to be shifted over a wider time range, strength value outcomes became more similar to those from the path shuffling method (figure 4*b*). However, degree values were most similar between the path shuffling method and intermediate time-shift range values (figure 4*a*).

Neither method failed to detect underlying social attraction where it existed (i.e. no false negatives) for the strong sociality scenarios we considered, for any of the simulations we tested. Even at low levels of social attraction, observed population means nearly always differed significantly from the randomizations (figures 3, 4: (ii),(iv),(vi)) and the measure that was most impacted by low social attraction was degree in the stable home range situation (electronic supplementary material, figure S3). However, the path shuffling method was more likely to detect false positives of sociality compared to the wrap-around method, especially when agents’ home ranges moved large distances in a directional manner (figure 5). The wrap-around method’s likelihood of detecting false positives increased as the sampling effort decreased (figure 5). Finally, both randomization methods detected sociality when we applied them to empirical data on flight interactions in a population of free-ranging vultures, though the wrap-around method was more similar to the vultures’ observed sociality values than the path shuffling method was, probably reflecting a better conservation of their movement continuity (figure 6).

### (a) Comparing path shuffling to the wrap-around randomization

Because both randomization methods disrupted the spatiotemporal synchrony of individuals that were moving together, they both succeeded in detecting underlying social attraction in all of the simulation scenarios (for *Z*-scores see the electronic supplementary material, tables S1–S3). This was owing in part to the relatively high level of social attraction we simulated. However, both methods still detected sociality even at much lower social weight thresholds, for both degree and strength (electronic supplementary material, figure S3). Overall, observed degree and strength values were more similar to the wrap-around randomization than to the path shuffling randomization because the wrap-around method retains temporal, as well as spatial, autocorrelation. By contrast, shuffling paths over the entire randomization period allows individuals to ‘teleport’ across their spatial range, allowing them to cover the spatial area more evenly in time.

The methods differed from each other the most when animals changed their activity areas directionally over the course of many days because the effect of ‘teleportation’ on the randomized trajectories was largest in this situation. Consider a case of two individuals who spend some multi-day portion of the study period preferring to associate with each other. If we apply the wrap-around method and end up shifting these two individuals' trajectories (relative to each other) by an amount less than their original period of association, then those two individuals will still have some co-occurrence (in time and space) in parts of their shifted trajectories. If, instead, we shuffle those trajectories across the entire study period, then there is no guarantee that the days when the two individuals interacted will stay adjacent; they could be reassigned to any date within the entire tracking period. If the individuals were changing their home ranges directionally, shuffling the days could also mean being separated far enough in *space* that interactions would be impossible.

The way that individuals use space is important for understanding how the two randomizations perform when there is no underlying social attraction. Notably, the path shuffling method over-predicted the population mean degree for the non-sociable agents whose home range centres changed locally (orange curve in figure 4*a*(iii)). Spiegel *et al*. [14] observed the same over-prediction of degree when they initially described the method. Key to this finding is the overlapping space use of the agents in the locally changing home ranges scenario (figure 2*b*(iii),(iv)). By applying path shuffling to these non-sociable agents, we increased the number of chance encounters with other unique individuals (i.e. increasing degree), which in the observed simulation are precluded by the temporal separation of the agents. By contrast, the wrap-around method maintains the internal spatiotemporal autocorrelation of each agent’s path, thereby retaining temporal isolation as a means of preventing encounters between certain individuals. Wrap-around-randomized individuals may interact with different unique individuals than in the observed simulation, but they will not necessarily encounter more individuals.

The distinction between the two methods is salient mostly for degree, which is a measure of the number of unique interaction partners, regardless of interaction duration or frequency. Neither the static home ranges scenario nor the directionally changing home ranges scenario resulted in larger than random degree values for the non-sociable simulation (figure 4*a*(i),(v)) because they lack the overlapping space use that characterizes the locally changing home range. This over-prediction of degree by the path shuffling method when animals have highly overlapping home ranges highlights the importance of constraining the extent to which we randomize movement trajectories—by constraining the shifting of each day based on its temporal sequence, we fix the problem of degree over-prediction and successfully capture the observed degree value (blue lines in figure 4*a*(iii)). This suggests that the wrap-around method may be especially important for biological questions about the number of unique individuals animals interact with (degree) and study systems in which overall space is shared between animals and social structure is maintained by temporal separation.

### (b) Effect of time shifts on wrap-around randomization performance

To apply the wrap-around randomization method, it is necessary to choose a time range that determines how far each individual’s trajectory can be shifted, as a proportion of the total tracking period. This limit represents a trade-off between test power and expected false-positive rate, and the selected time-shift range should reflect the biology of the system and the relevant rate of change in individuals’ movement and space use. While the wrap-around and shuffling methods randomize paths by days, to avoid decoupling activities that occur at different times of day, there could be biological systems and research questions in which shorter or longer time units would be more appropriate. Restricting time-shifts to very small ranges (e.g. 2 days in our simulations) results in randomized trajectories that are very similar to the observed data: each individual has only two possible starting days, so each individual will remain correlated with half of the others in the population—thus reducing the statistical power and potentially leading to a higher false-negative rate. Indeed, we found that when social attraction was very slight, the likelihood of a false-negative result was higher for the highly restricted wrap-around method than for either the path shuffling method or wrap-around with a wider range (electronic supplementary material, figure S3). At the other extreme, letting trajectories shift over the entire tracking period effectively allows any first day of a trajectory to fall on any other day, bringing the adjacent days in the trajectory along with it. By increasing the number of possible start dates, the largest possible time-shift range would minimize the chance of any two individuals being exactly aligned with each other. Interestingly, it did not take much time-shifting to disrupt the social structure and detect sociality. Even the shortest time-shifts still returned results that were significantly different from the observed values (figure 4*a*,*b*(ii),(iv),(vi)), including when the randomization method was applied to empirical data (figure 6). A randomization that changes the data minimally while still distinguishing between the causal factors of interest is desirable because it avoids problems associated with longer time scales, such as changing environmental conditions.

The time-shift size that is required for detecting sociality when using the wrap-around method can depend on the duration of social interactions relative to the duration of the time-shift units. In our simulation, social interactions were brief, while trajectories were shifted backward and forward by entire days. Therefore, even shifts of 1 or 2 days were sufficient to disrupt social associations. This was especially true for our analysis of vulture data, in which co-flight interactions could be very brief. If the shifting and interaction durations are on similar time scales, one might be able to examine different time-shift ranges to determine the duration of a biologically meaningful interaction. Such an approach would be similar to Whitehead’s [52] lagged association-rate analysis. Further analysis of the wrap-around method can highlight its use for identifying the time scale over which social interactions occur and persist. Another relevant consideration is how social interactions are defined. We used a simple co-location definition of social interactions in our simulations, in which being in close proximity during one time step was sufficient for two individuals to be considered interacting. This may or may not be biologically accurate for a given study system. The definition of a social interaction may change the extent of the difference between observed and randomized networks, with the co-movement definition excluding brief ‘path-crossings’ from consideration and therefore potentially causing randomizations to differ even more from observed networks than under a co-location interaction definition. A reanalysis of our simulated social networks with a co-movement definition of social interactions yielded qualitatively similar results for all simulations (electronic supplementary material, figures S5), but we encourage further examination of the effect of interaction definitions on randomization results.

In the version of path shuffling we used for comparison, days are randomized over the entire tracking period (any day can be assigned to any other day). This approach is most similar to conducting the wrap-around randomization with the largest possible time-shift range. However, they are not identical because even when trajectories shift over many days, some pairs of individuals will, by chance, have *relative* shifts that are small enough to maintain more social interactions than they would when using the path shuffling method. Path shuffling can be alternatively implemented with restricted time windows, as discussed by Spiegel *et al*. [14]. For instance, one could choose to break a 100 day season into 10 day segments and randomly shuffle the days only within each segment, which would preserve some of the spatiotemporal autocorrelation that is lost when days are shuffled across all 100 days. Note that the default implementation of the path shuffling method in the R package ‘spatsoc’ [13] randomizes across the entire tracking period, but the user can break the tracking period into shorter segments. Spiegel *et al*. [14] conducted a thorough analysis of the effect of the time-window size on the performance of the path shuffling approach. Here, we shuffle paths over the entire tracking period, because this is how the path shuffling method has been implemented in most cases [29–31] and because even the smallest time-shift ranges of the wrap-around method allow for the possibility of interchanging trajectory segments at either end of the tracking period. Future work could examine how the time window for path shuffling and the time-shift range of the wrap-around method correspond to each other. For both methods, shorter time-shift ranges are recommended if the typical duration of a social interaction and the rate of environmental change are shorter.

From our comparison of just one path shuffling time-window to many time-shifts of the wrap-around method, we see that the relationship between the two methods differs depending on the network measure examined (degree or strength). When considering strength, the path shuffling method was most similar to the wrap-around method with the widest possible time-shift range (lightest blue lines, figure 4*b*). This is as expected, considering that both of these methods can randomize any day to any other. However, we did not observe the same pattern for degree—instead, path shuffling was most similar to wrap-around with an intermediate time-shift range (medium blue lines, figure 4*a*). This pattern is explained by the conceptual difference between degree (which counts interaction partners, no matter how brief the interaction) and strength (which considers interaction frequency). Allowing trajectories to shift by a large number of days increases the number of pairs of individuals whose trajectories will be decoupled to the point that they will never interact (reducing their degree), while smaller shifts will preserve at least some chances for them to encounter each other. By contrast, path shuffling creates new, brief interactions between individuals that would never have met otherwise. Those fleeting interactions are sufficient to increase degree (because even a single interaction counts, whereas the effect on strength is minute). In this way, degree can reach beyond its value in the observed data—as discussed below. Meanwhile, strength does not show the same effect, because it is agnostic to the identities of the interacting partners. Therefore, in selecting a randomization method and a time-shift range, it is important to consider how randomization methods may behave differently depending on the network measure that is being examined. Depending on the research question, there are many other network measures, in addition to the two commonly used ones we considered here. The difference observed here between degree and strength suggests a potentially general pattern that social network measures that are sensitive to extreme events (like degree) will show more differences across randomization methods compared to those that are the cumulative effect of many connections (like strength).

### (c) Sampling frequency and false positives

While both randomization methods succeeded in detecting social attraction in our sociable agents simulations, the changing home ranges sometimes led to detecting social attraction or avoidance when it did not exist (non-sociable agents). A false-positive signal of social avoidance was detected only once by the path shuffling method, for degree in the locally changing home ranges scenario (orange curve in figure 4*a*(iii)) because of individuals' overlapping space use, as discussed above. A false-positive signal of social attraction was detected for both degree and strength in the directionally changing home ranges scenario, for both path shuffling and the long (but not the shortest) time-shift ranges in the wrap-around method (figure 4*a,b*(v),(vi)). When animals in the simulation had directionally changing home ranges, they all started relatively close to each other and then radiated outwards and away from the starting position and from each other. When trajectories are wrapped around, the end of the trajectory is linked to its start, allowing individuals that were too far from each other at the end of the simulation to interact with each other. Only the smallest time-shifts maintain the overall spatial structure of the population, resulting in the lowest rates of false positives when animals move in a directional manner. Finally, the size of the time-shift range had opposite effects on the likelihood of the wrap-around method to detect false positives in each of these scenarios because one of them is a case of the observed being artificially above the expected (false positive for attraction in the directionally-changing home ranges scenario, figure 5*e,f*), while the other is a case of the observed being artificially below the expected (false positive for avoidance in the locally-changing home ranges scenario, figure 5*c,d*).

The rate of false positives increased as trajectory data was downsampled for the wrap-around method, especially when the home range centre of the agents moved locally (figure 5*c,d*). As data become more sparser, error rate increases, just as with any sampling method, not only of movement data. An increase in error rate results in higher rates of type I errors, i.e. more false positives, using any statistical approach. Both randomization methods had some false-positive rates at very low sampling rates (when only 10% of the data was sampled) for the locally changing home ranges scenarios, suggesting that as information about the movement pattern becomes less complete, the difference in performance of the two methods is smaller when animals have locally moving home ranges. Interestingly, sampling frequency did not have as much of an effect on the likelihood of reporting a false positive using the wrap-around method with small time-shifts, in the directionally changing or static home ranges scenarios (figure 5*a,b,e,f*). This lower impact of sample size on false positives might be owing to the very localized, or the large ranging movements of the individuals that are both still captured relatively well within the time scale of the randomization with downsampled movement data. Thus, the way in which animals move around in their environment could influence the impact that sampling effort has on the ability to test hypotheses about the underlying causes of sociality.

While we investigated the performance of our proposed method when the simulated data were downsampled, we did not explore the effect of irregular missing observations or missing individuals. Furthermore, in animal tracking studies, it is common for individuals to be tracked over different portions of the overall study period. Future work could explore how the wrap-around method performs when individuals are not tracked for the entire study duration. There are two ways to adjust the wrap-around method to datasets with individuals differing in sampling duration: (i) wrap each individual’s trajectory around itself, retaining the distinction between tracked and untracked days (the approach we took for the vulture data); and (ii) assuming that individuals are present in the population even when they are not tracked, shifting trajectories throughout the entire tracking period, including days in which an individual was not observed. The potential effects of each of these wrap-around versions on the sensitivity and specificity of the wrap-around method should be explored.

### (d) Using path randomizations to determine the causes of sociality in free-ranging vultures

Both randomization methods detected social attraction when applied to empirical data from a population of free-ranging vultures, rejecting the hypothesis that vultures interact at random and supporting the common understanding that vulture interactions emerge from conspecific attraction, beyond the influence of spatial constraints alone (figure 6; [45,48]). Vultures do change their centres of activity from day to day, but not to the same extent as our directionally changing home ranges simulation scenario (see the electronic supplementary material, figure S1 for examples of vulture trajectories). Their movements are highly spatially overlapping over the course of the season; conceptually, they might fall somewhere between our static and locally changing home range scenarios (electronic supplementary material, figure S1). Congruent with our findings from the simulation data, both randomization methods were significantly different from the observed values for degree and strength, reflecting vultures' strong sociality. Indeed in the simulations, both methods, but especially the wrap-around method with a wide time-shift range, succeeded in detecting social attraction even when it was quite weak (electronic supplementary material, figure S3), and can therefore be used on species with low rates of sociality. As with the simulated data, the wrap-around method produced results that were more similar to observed values than the path shuffling method, because wrap-around maintains the spatial as well as the temporal autocorrelation of the data. Wrap-around randomization detected clear deviations from random for both degree and strength even when time-shifts were limited to a very small range (figure 6*b,d*). Future work should examine the effect of the absolute number of days, rather than just the length of the time-shift range relative to the length of the entire sampling period, on the difference between observed and expected values. Furthermore, having rejected the hypothesis that vultures interact at random after accounting for their movement patterns, we anticipate further work examining the relative importance of spatial attractors and social preferences in shaping their social behaviour.

Both randomization methods face challenges when the landscape itself changes frequently. The case study of the vultures provides particular examples for potential pitfalls related to changing resources. Vulture flight movements are strongly affected by the location and strength of thermal and orographic uplift, which are temporally (and, in the case of thermal uplift, spatially) variable [53–55]. The carcasses they feed on are ephemeral and also highly variable in space and time [56,57]. A particular carcass may be visited for a few consecutive days, serving as a local attractor. The goal of randomizations is to decouple the temporal synchrony of pairs of individuals while keeping each individual’s movement tied to the geographical space in which it is moving. However, when key features of the landscape (such as uplift, ephemeral food, etc.) are themselves changing in time, randomizing trajectories will also decouple individuals' movements from the times when the resources were present. Such decoupling could generate false-positive signals of social attraction when animals are aggregating around fleeting resources rather than being attracted to each other [58,59]. Adjusting the time-shift range (for wrap-around) and time window (for path shuffling) to suit the temporal scale over which resources change could minimize the impact of this decoupling. We have shown that even time-shifts of a day or two are sufficient for detecting significant differences between observed and randomized data. If a particular resource tends to change approximately every 3–5 days, then shifting trajectories by 1–2 days could minimize the decoupling of individuals from ephemeral resources while still disrupting temporal synchrony among individuals. Still, disentangling the influences of a changing landscape on animal movements—and therefore on their social interactions—from the effects of their social preferences, and our ability to detect them, and other potential drivers of sociality, remains a significant challenge that is not adequately addressed by some of the current methods [8].

## 5. Conclusions

Uncovering the underlying causes of sociality is often addressed by comparing observed data to expected values from randomizations that decouple parameters of interest, like spatial and social processes. The way in which randomizations are designed therefore impacts the ability to make inferences about underlying biological processes. We show that randomization approaches that retain spatiotemporal attributes of movement paths in different ways differ in their ability to detect sociality for certain types of movement patterns. Specifically, the wrap-around method that we proposed here outperforms the commonly used path shuffling approach by: (i) producing randomizations that are more similar to the observed data, thus creating values that are more biologically feasible; and (ii) reducing the false-positive detection rate when animals shift their home ranges. Thus, the wrap-around method is applicable to more types of animal movements than path shuffling and is especially more suitable for systems in which space is shared between animals but social structure is maintained by temporal separation. We note however that regardless of which method is used, if the density of individuals changes over time (e.g. because individuals spread out in certain seasons) the false-positive rates will increase if the time scale of seasonal effects is not considered. It would be further interesting to examine how the two randomization approaches perform when populations are composed of individuals that differ substantially in their movement patterns. Considering the use of the wrap-around method to a broad range of movement types, its lower error rate, and increased biological realism, we recommend implementing it in the highly used R package spatsoc [13] and using it over the path shuffling method—especially when animals do not return to the same location each night.

## Data Availability

R code, including for running the simulations and all analyses, can be found in the GitHub repository [60]. Vulture data for summer 2022, along with a shapefile of roost sites, are available in the electronic supplementary material. Simulation data is reproducible from the code contained in the GitHub repository. Supplementary material is available online [61].
